# Sickness of Prof. Julius F. Miner

**Published:** 1880-10

**Authors:** 


### SICKNESS OF PROF. JULIUS F. MINER.

We regret to announce the serious and alarming indisposition of Dr. Miner, who now lies prostrate in a condition to cause the deepest anxiety among the profession in this city. Dr. Miner's health has been impaired for several years, but until within a few weeks, he has been able to attend to his extensive practice, and to perform even the severer surgical operations, which his acknowledged ability and wide reputation commanded. In the early part of September he was stricken with right hemiplegia, accompanied with great prostration of the vital powers. At one time his life was despaired of, but we are permitted to state, through the courtesy of Profs. White and Rochester, that a gradual improvement has taken place in his paralytic symptoms, and the indications for his recovery are more favorable than a week ago.

The deepest interest and anxiety prevail in professional circles, both here and elsewhere, for the distinguished patient whose life now hangs upon so uncertain a tenure, and we earnestly trust that the strong and indomitable will, which has mastered so many difficulties in the past, may bear him safely through the severe ordeal of his present sickness, and spare his life for future work in the profession he has so much adorned.

